# The Management of an Intraperitoneal Leak Following Transgastric Stenting of a Pancreatic Pseudocyst

**DOI:** 10.7759/cureus.7236

**Published:** 2020-03-10

**Authors:** Alexander Mimery, Minh Pham, Willy Kok Wai Low, Amitabha Das, Kheman Rajkomar

**Affiliations:** 1 Surgery, Gladstone Hospital, Gladstone, AUS; 2 Surgery, Bankstown Hospital, Sydney, AUS; 3 General Surgery, Albany Regional Hospital, Albany, AUS

**Keywords:** case report, general surgery, hepatopancreatobiliary, pseudocyst, transgastric stenting

## Abstract

The traditional management of pancreatic pseudocyst (PP) is surgical drainage; however, there is significant morbidity associated with this approach. An endoscopic ultrasound (EUS)-guided transgastric endoscopic approach is preferred if there is favourable access to the PP.

This case report describes a rare complication of an EUS-guided transgastric drainage of a PP secondary to a suboptimally positioned stent. Significant soiling of the peritoneal cavity by pancreatic juices and gastric contents occurred due to leakage around the stent puncture sites. A novel technique using an infant feeding tube is described to inflate the collapsed PP and facilitate definitive surgical cystogastrostomy.

A literature review and discussion surrounding the safety of endoscopic decompression and the type of stent utilised is also presented.

## Introduction

Pancreatic fluid collections are common sequela of pancreatitis. An acute peripancreatic fluid collection is a non-encapsulated fluid collection that develops within four weeks of pancreatitis onset [1]. A pancreatic pseudocyst (PP) is an encapsulated pancreatic fluid collection that develops after four weeks of onset. The prevalence of PP has been described in literature to range between 6% and 18.5% in acute pancreatitis and 20% and 40% in chronic pancreatitis [2-4].

The majority of PPs do not require treatment and will regress spontaneously. Small pseudocysts (<5 cm) are expected to resolve spontaneously in over 90% of cases [3]. Intervention is indicated for PP that becomes infected, or if there is a mass effect exerted on surrounding structures. The traditional management of PP is surgical drainage (either open or laparoscopic); however, there is significant morbidity associated with this approach. An endoscopic ultrasound (EUS)-guided transgastric endoscopic approach is preferred if there is favourable access to the pseudocyst [5].

EUS drainage of a PP may be performed by a transpapillary or transmural (transgastric/transduodenal) approach. General criteria for this approach include a mature fluid collection, close proximity to the stomach or small bowel, PP greater than 6 cm, and the absence of a pseudoaneurysm (unless embolised prior) [6]. Appropriate preinterventional imaging is essential to determine if these preconditions have been met. A transpapillary approach is indicated for PP that communicates with the pancreatic duct. A transmural approach involves the development of a tract between the visceral lumen and the PP, with subsequent balloon dilatation and the placement of a stent. The overall technical success rate of endoscopic drainage is over 90%, with a 70%-80% resolution rate and a 10%-15% recurrence rate [7-10]. Complications of endoscopic drainage may be acute (bleeding, perforation) or delayed (infection, stent occlusion), and is sometimes catastrophic [10]. Inadvertent perforation and peritoneal breach during the drainage process will lead to seepage of pancreatic and gastric juices into the peritoneal cavity, which can be fatal if unrecognised and not salvaged promptly via surgery. During surgical intervention, a definitive plan to decompress the pseudocyst is necessary as future interventions are limited. Often the decompressed PP can be difficult to locate, especially if it is thick walled, recurrent and in the setting of sepsis. This case report describes a novel and simple strategy of inflating the PP to facilitate a safe surgical cystogastrostomy.

## Case presentation

A 68-year-old male presented to the outpatient endoscopy unit for a repeat EUS drainage of a PP.

The patient has a background history of necrotising gallstone pancreatitis that was treated supportively. A subsequent laparoscopic cholecystectomy was performed. Unfortunately, he developed a PP that required EUS-guided drainage approximately 12 months following his index presentation. A surveillance CT abdomen demonstrated a large recurrence of his PP (approximately 13 cm in largest diameter); thus, a decision for repeat drainage was made. His other comorbidities include chronic obstructive pulmonary disorder, hypertension, and a solitary kidney (donor nephrectomy).

**Figure 1:**
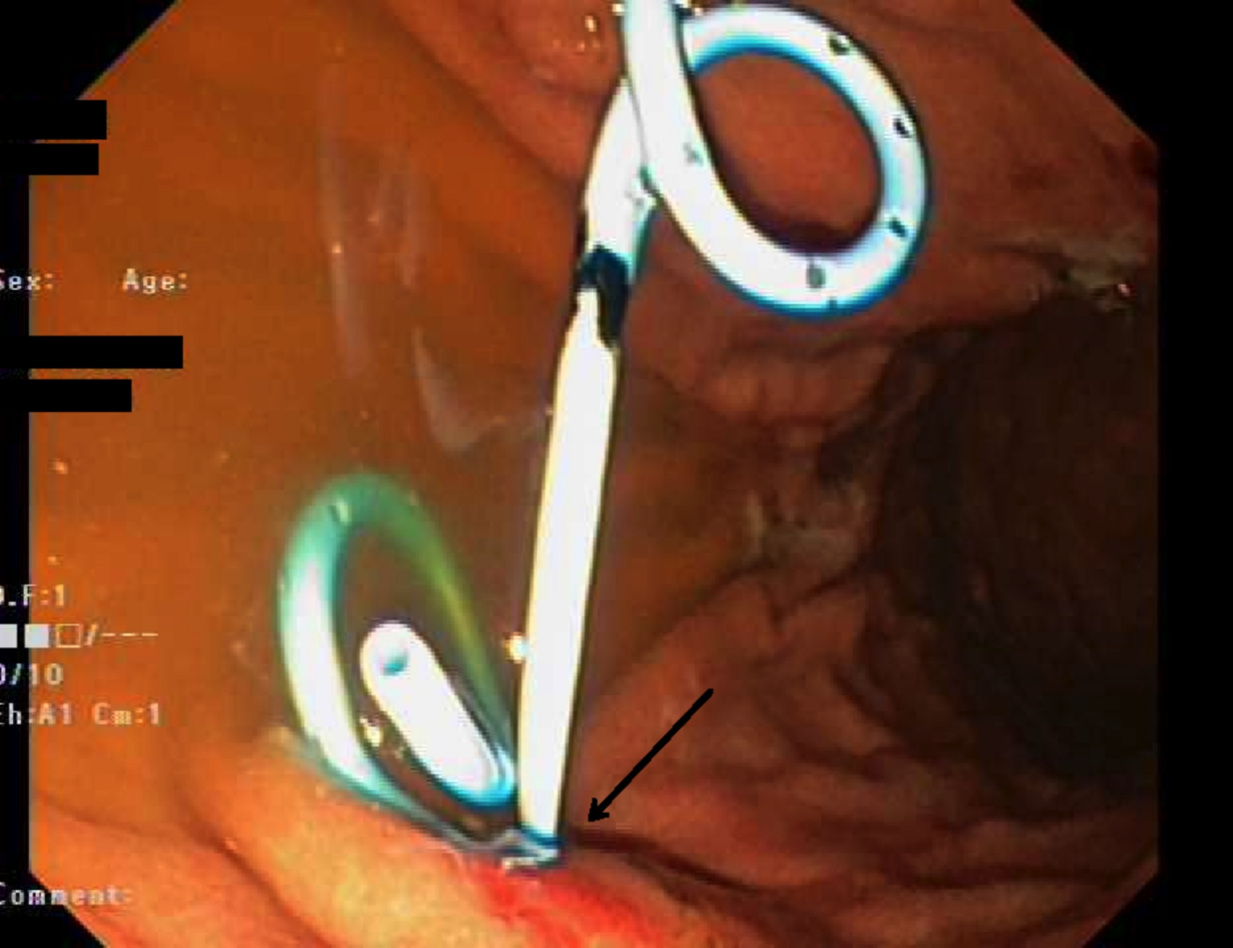
An endoscopic image demonstrating the final position of the two pigtail plastic drains inserted after EUS-guided drainage. The arrow demonstrates the site where the stent punctures through the gastric wall. EUS, endoscopic ultrasound

A linear echoendoscope was advanced to the second part of the duodenum. External compression of the posterior gastric wall was observed endoscopically. Endosonographically, the PP measured 13 x 10 cm and was punctured using a 19-gauge access needle. Approximately 10 ml of clear fluid was aspirated, and then injected with 20 ml of contrast. A 450-mm Jagwire was passed through the needle into the pseudocyst under fluoroscopy. The access needle was exchanged for a cystotome. A cystpogastrostomy was performed using the cystotome, and a second 450-mm Jagwire was inserted. The cystotome was removed, and two 7 French plastic double pigtail stents were introduced into the PP over the two guidewires under direct fluoroscopy. Both stents appeared to have been positioned satisfactorily, and were draining clear fluid into the stomach (Figure 1).

**Figure 2:**
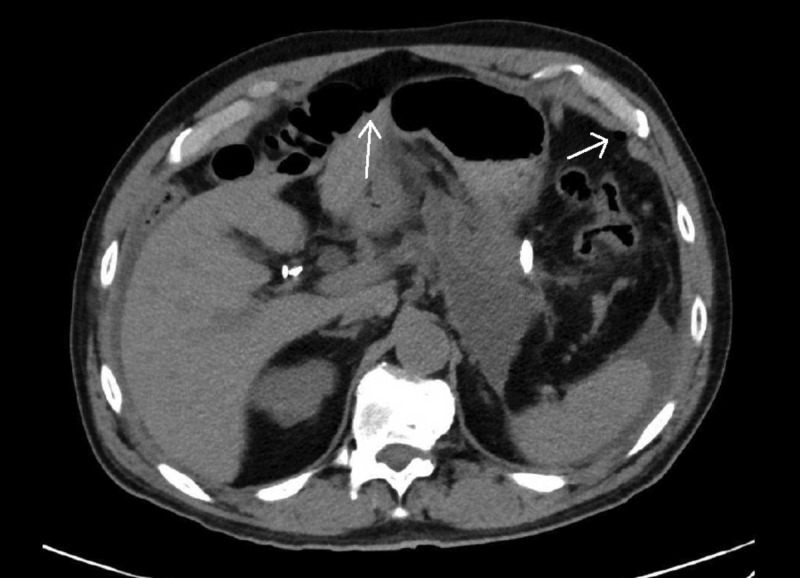
A post-EUS drainage CT abdomen (non-contrast) axial view demonstrating pneumoperitoneum (arrows) concerning for a hollow viscus perforation. EUS, endoscopic ultrasound

The patient developed severe abdominal pain in the recovery bay. An urgent CT abdomen demonstrated free fluid and gas in the upper abdomen especially anterior to the liver (Figure 2). A decision was made to proceed to the operating theatre given concerns for a perforated hollow viscus.

**Figure 3:**
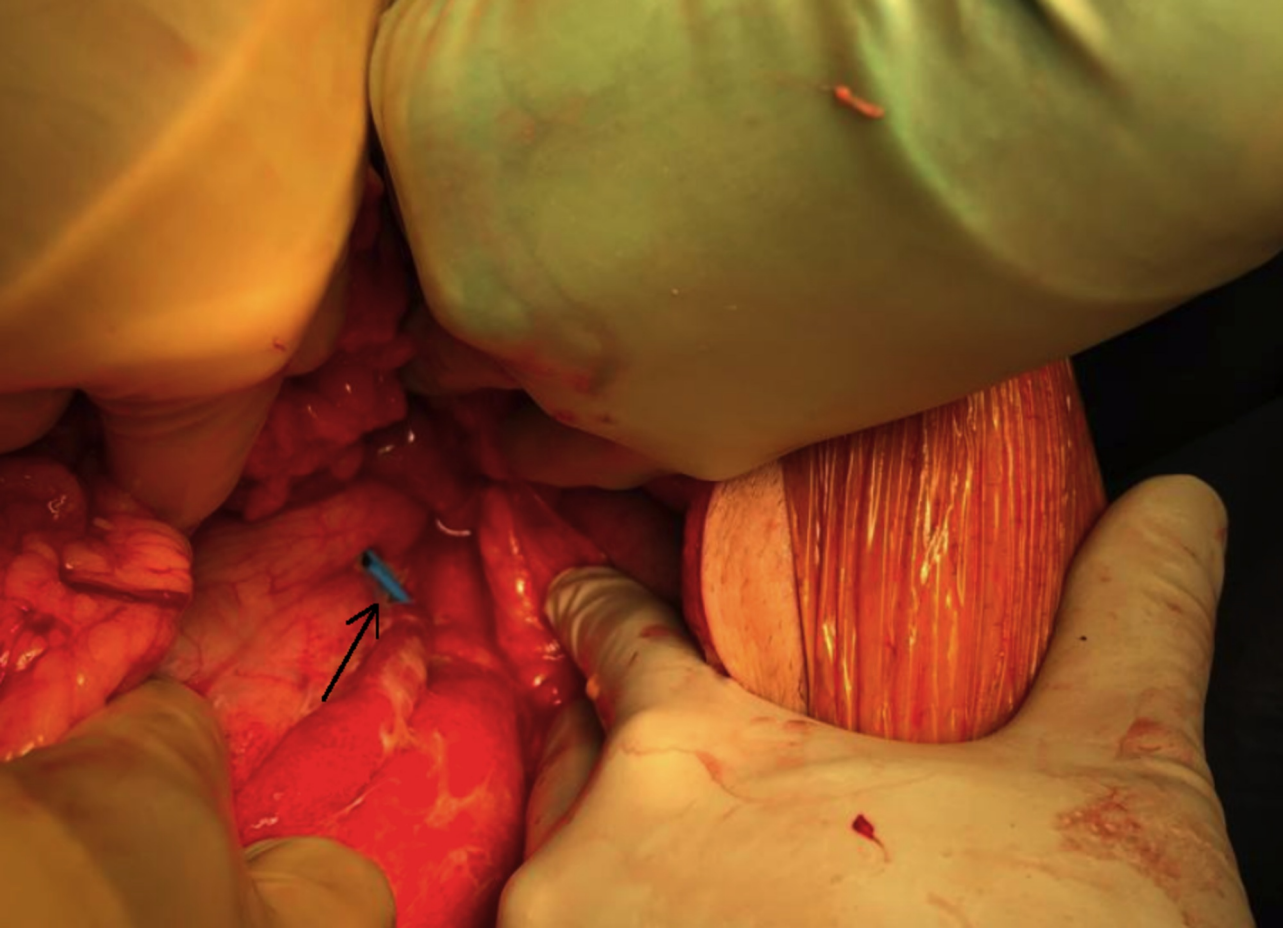
An intraoperative image demonstrating the intraperitoneal path of the stent (arrow) between the stomach and the pancreatic pseudocyst.

A midline laparotomy was performed. Significant amounts of gastric and pancreatic juices were found throughout the entire peritoneal cavity and evacuated. The gastrocolic ligament was taken down with the harmonic scalpel to allow access to the pancreas. The cystogastrostomy tract and stents were found to have inadvertently entered the peritoneal cavity (Figure 3), and thus were removed.

**Figure 4:**
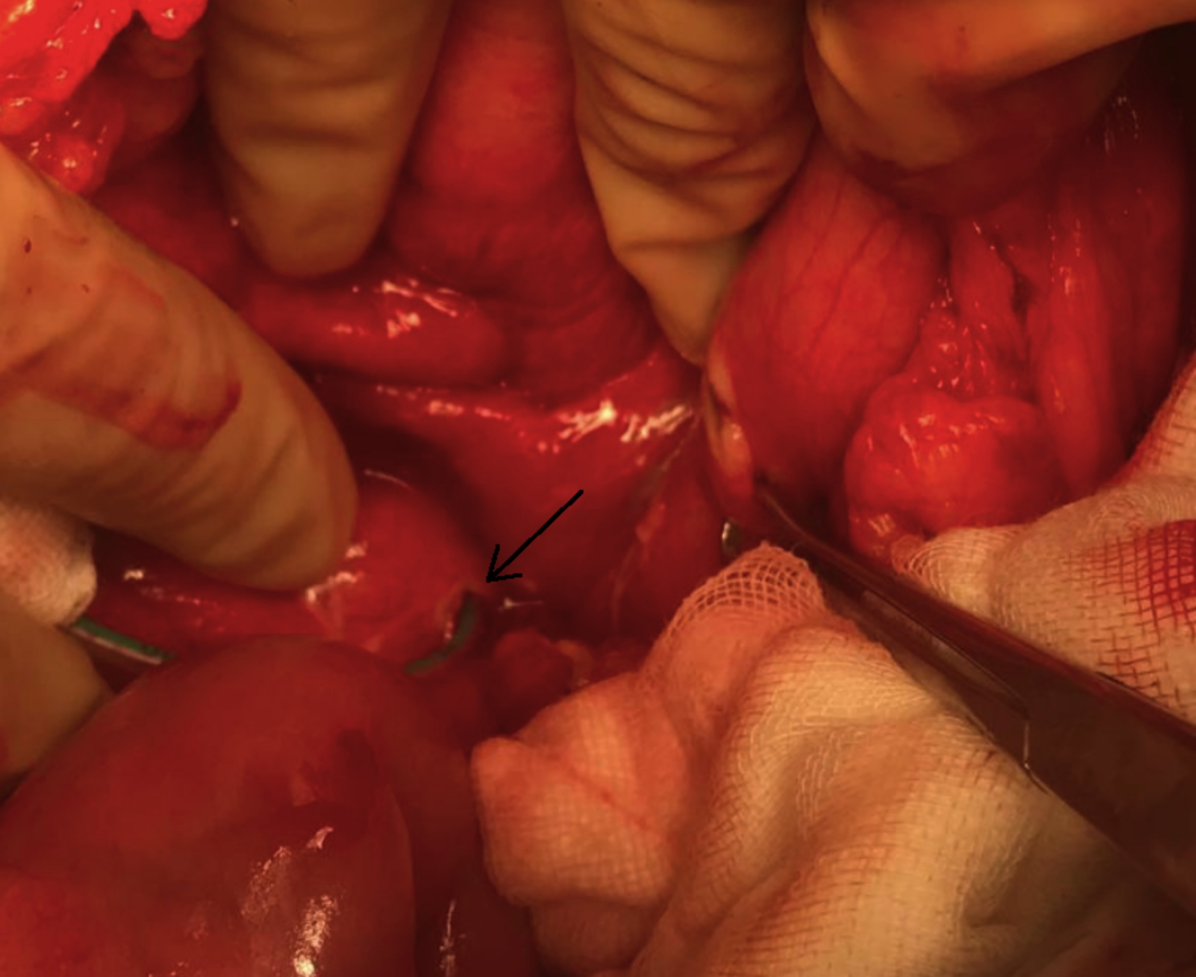
An intraoperative image demonstrating the insertion of an 8 French infant feeding tube into the pancreatic pseudocyst (arrow).

A decision was made to surgically decompress the PP given the high likelihood of recurrence. An anterior longitudinal gastrotomy was performed. There was difficulty locating the PP through the posterior stomach wall as it had collapsed, having spilled its contents into abdominal cavity. The PP cavity was not identifiable despite using an intraoperative ultrasound (IOUS). An 8-French infant feeding tube was inserted through the peritoneal defect caused by the transgastric stent, and placed into the PP cavity. The feeding tube was subsequently injected with saline (Figure 4).

**Figure 5:**
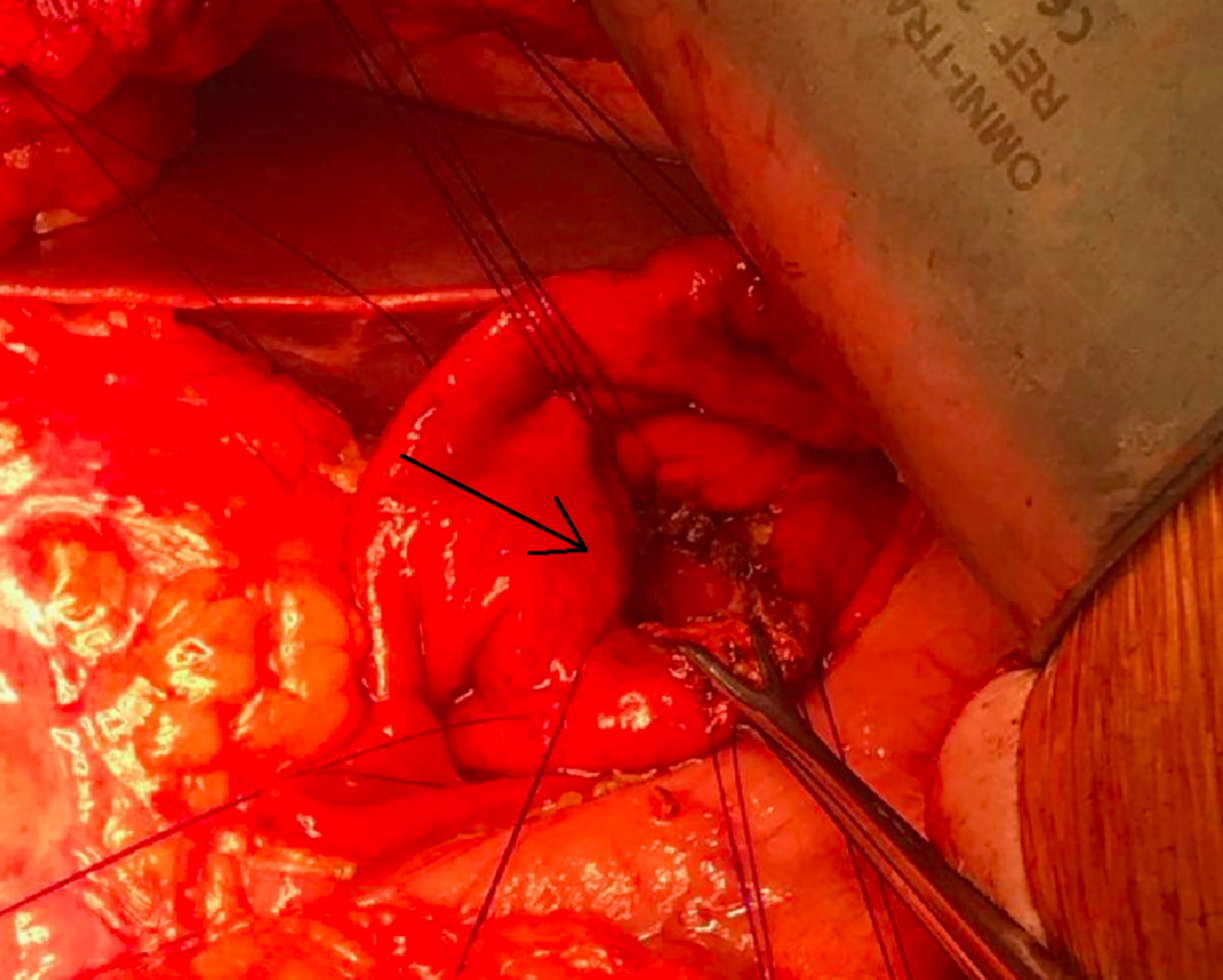
An intraoperative image demonstrating the construction of a surgical cystogastrostomy (arrow).

A subsequent attempt at transgastric localisation of the PP was successful using IOUS given the re-expansion of the PP. A seeking needle was then used to confirm the location of the PP. A 45-mm Endo-GIA (Medtronic plc, Minneapolis, MN) stapler was used to complete the cystogastrostomy, and the edges oversewn with 3-0 prolene (Figure 5). The anterior gastrotomy wound was closed with 3-0 PDS. The PP defect and corresponding gastrotomy defect were closed with 3-0 PDS. A feeding jejunostomy was fashioned. The abdomen was washed with saline and two drains placed: one on the anterior stomach and one on the closed pseudocyst defect.

The patient recovered well postoperatively. A CT contrast swallow was organised on day 10 after the operation, which demonstrated no evidence of any leak. The abdominal drains were removed after drain amylase levels were found to be normal. The patient was initially supported with postpyloric feeds through his feeding jejunostomy, and then slowly transitioned back into a regular diet. He was discharged from hospital two weeks after his operation. He remains free from any recurrence 13 months after his surgery.

## Discussion

**Figure 6:**
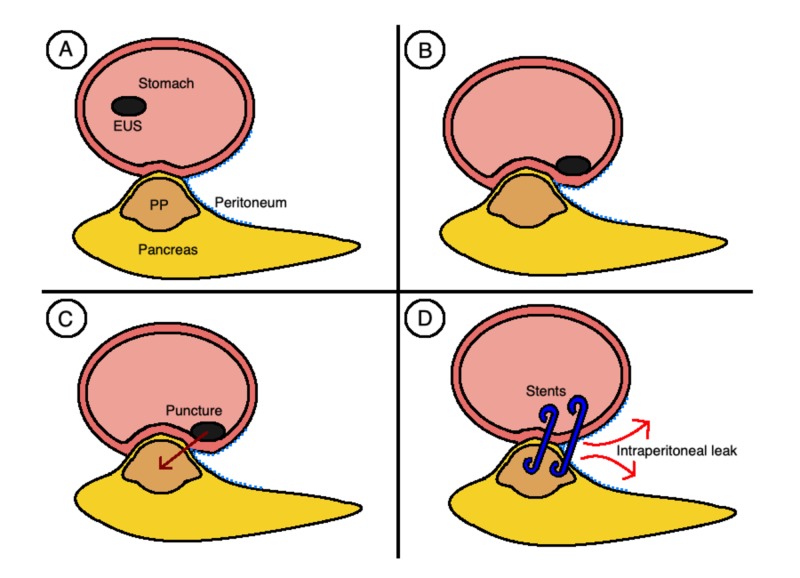
A diagram demonstrating how accentuation of the degree of stomach to PP apposition caused by the EUS probe against the posterior gastric wall resulted in an intraperitoneal leak. EUS, endoscopic ultrasound; PP, pancreatic pseudocyst

This case report describes a rare complication of an EUS-guided transgastric drainage of a PP. It was suspected that the fundamental cause of this complication was a suboptimally positioned stent that freely traversed through the peritoneal cavity. The space between the posterior wall of the stomach and the posterior peritoneal reflection is often obliterated given the mass effect of the PP. Therefore, the stent should traverse through the stomach wall, into the retroperitoneum, and directly into the PP through the shortest route possible. In this case, the EUS probe pressure on the posterior gastric wall falsely accentuated the degree of apposition between the PP and the stomach. This resulted in the stent being placed too laterally. Consequently, when the stomach wall returned to its normal anatomical position postprocedure, it dragged the stent anteriorly thus exposing a segment freely within the peritoneal cavity (Figure 6). Significant soiling of the peritoneal cavity by pancreatic juices and gastric contents occurred due to leakage around the stent puncture sites.

Endoscopic transmural drainage of PP is effective and safe in suitably located collections that are well apposed to the posterior gastric wall. An upfront surgical transgastric approach can have significant morbidity compared to an endoscopic approach. Furthermore, in a randomised trial, endoscopic cystogastrostomy was found to be associated with lower cost, hospital stay, and better quality of life than a surgical approach [11]. Sterile PPs (such as in the case described above) are easier to drain endoscopically, and have a good complication profile with rates of bleeding/perforation being about 5% [11,12]. The complication rate increases significantly (up to 30%) in infected pseudocysts [12].

A systematic review had shown no superiority in PP resolution and adverse events when metal stent or plastic stent is used in pancreatic fluid collections [13]. However, a study looking specifically at PP drainage found that the use of plastic stent was associated with more adverse events than fully covered self-expanding metal stent [14].

More recently, lumen apposing metal stents (LAMS) have been found to be equivalent, if not better, than plastic stents in pancreatic fluid collections, in terms of resolution and complications [15,16]. This may attributable to their flanged design and increased luminal size (10-15 mm), which limits migration and achieves a success rate of more than 90% in some series [17].

So, why not use metal stents all the time? Firstly, the quality of the evidence is not very strong. Studies are mainly of cohort type and heterogeneous with respect to type of stents used or patient selection, with mostly low patient numbers [13,16]. Secondly, the published studies pertaining to the use to metal stents, especially LAMS, have been undertaken in cases of pancreatic fluid collections, not just PP. Thirdly, metal stenting is associated with increased hospital costs compared to plastic stenting, by up to US$ 40,000 in some series [18]. Finally, there are also complications associated with metal stents. They can cause local erosions, and may result in bleeding. Intraperitoneal penetration associated with the maldeployment of LAMS, requiring an emergent open cystogastrostomy, has also been described in literature [19].

A novel technique is described in this report to assist in the localisation of a collapsed PP. Several attempts to localise the PP clinically and using IOUS were unsuccessful as the majority of its contents had spilled into the peritoneal cavity. The difficult localisation of a thick-walled recurrent pseudocyst that has been decompressed makes the creation of a surgical cystogastrostomy difficult and hazardous. There is a serious risk of bleeding if the correct window through a thick vascularised gastric-pseudocyst wall is not identified. Additionally, a blind puncture runs the risk of a retroperitoneal vascular injury, such as splenic vessels. Inflation of the pseudocyst with fluid via the feeding tube is safe and easy and enables the surgeon to target his cystogastrostomy appropriately. To the authors’ knowledge, this strategy has never been described in the medical literature.

Although an endoscopic cystogastrostomy of a simple, large, mature pseudocyst that is well apposed to the gastric wall is technically possible with minimal morbidity, several steps must be considered to avoid an inadvertent perforation. The endoscopist must be aware that probe pressure on the gastric wall may exaggerate the degree of apposition of the PP to the stomach. Secondly, the pseudocyst stent deployment must be performed with care. The fluoroscopic images should be carefully assessed during the procedure to make sure that the cyst has been well accessed and also that the stent is well positioned. 

## Conclusions

Careful access to the PP is critical, even with EUS guidance, to prevent inadvertent intraperitoneal perforation. Close postprocedural observation is mandatory to detect any early procedural related complications, which may be fatal if unrecognised. The authors recommend a multidisciplinary discussion when planning drainage. Furthermore, it should also be performed in a facility where pancreatobiliary surgical services are readily available. We demonstrate that a decompressed thick-walled pseudocyst can be reinflated with an infant feeding tube to facilitate a safe surgical cystogastrostomy.
